# Molecular detection of class 1, 2 and 3 integrons and some antimicrobial resistance genes in *Salmonella* Infantis isolates

**Published:** 2018-04

**Authors:** Fariba Asgharpour, Seyed Mahmoud, Amin Marashi, Zahra Moulana

**Affiliations:** 1 Faculty of Para-Medicine, Babol University of Medical Sciences, Babol, Iran; 2 Department of Microbiology and Immunology, Qazvin University of Medical Sciences, Qazvin, Iran; 3 Infectious Diseases and Tropical Medicine Research Center, Babol University of Medical Sciences, Babol, Iran

**Keywords:** Integrons, *Salmonella infantis*, Multidrug resistance, Poultry

## Abstract

**Background and Objectives:**

Multidrug resistant *Salmonella* strains have been observed around the world in recent years. Many mechanisms contribute to the spread of antimicrobial resistance genes. This study aimed at determining the distribution and transmission of class 1, 2 and 3 integrons among MDR *Salmonella* isolates collected from a selection of chicken broilers in the north of Iran.

**Materials and Methods:**

PCR assays were used to detect genes for tetracyclines (*tetA, tetB* and *tetG*), chloramphenicol (*cat1* and *floR*), and streptomycin (*strA*). Also, the presence of class 1, 2 and 3 integrons in all MDR isolates was evaluated using specific primers for the integrase genes of integrons *intI1, intI2* and *intI3*.

**Results:**

Class 1, 2 and 3 integrons were present in 36%, 42% and 4% of the MDR isolates, respectively. Out of the tetracyclines resistant isolates, 47 (100%) and 5 (10.6%) carried *tetA, tetB* genes, respectively, while no isolate was positive for the *tetG* gene. All 36 chloramphenicol-resistant strains carried *floR* and *cat1* genes. Nine (18%) *Salmonella* Infantis isolates harbored the *strA* gene, conferring resistance to sterptomycin.

**Conclusion:**

This study found a high frequency of antimicrobial resistance genes among *Salmonella* isolates; therefore, management strategies are needed to prevent food-borne diseases caused by MDR *Salmonella* from food supplies.

## INTRODUCTION

*Salmonella enterica* subsp. *enterica* serovar Infantis can cause a variety of infections in humans and numerous animal species (1, 2). Nowadays, the distribution of multidrug-resistant (MDR) bacteria including *Salmonella* Infantis has become a global concern (3). Chickens are well known reservoirs for the transfer of antimicrobial resistant bacteria and antimicrobial resistance genes in many countries (4–8). Many studies show high rates of antimicrobial resistance among *S*. Infantis isolates in poultry, mainly to ampicillin, nalidixic acid, streptomycin, sulfonamides, and tetracyclines (9). The main mechanism responsible for the increase in antimicrobial resistance is horizontal gene transfer through mobile genetics elements, such as plasmids, transposons, and integrons (10). Integrons are DNA elements that can transfer antibiotic resistance genes between bacteria (11). Three classes of integrons have been identified, *intI1, intI2* and *intI3*, which are reported to be associated with antibiotic resistance genes (12). Class 1 integrons have been strongly associated with encoding over 130 resistance gene cassettes. However, only 6 cassettes have been identified in class 2 integrons. Limited diversity is also observed in class 3 integrons in the literature and the GenBank database (6, 10, 13). Although many investigations have been performed on the prevalence of integrons in food-borne MDR *Salmonella* isolates around the world in recent years, there is limited information on the distribution of class 1, 2 and 3 integrons among food transmitted pathogens and their association with MDR phenotypes in Iran.

Due to the increasing prevalence of antibiotic resistant *Salmonella* isolates, it is important to determine the transfer route of genetic elements, such as integrons and plasmids, which disseminate antibiotic resistance genes through horizontal or vertical transfer. The route of transfer plays an important role in the evolution of multidrug resistance and shows that *Salmonella* antibiotic susceptibility does not have a homogeneous distribution, neither geographical nor temporal. Surveillance programs are needed to monitor the evolution of antibiotic resistance and presence of mobile genetic elements. This study aimed at determining the distribution and transmission of class 1, 2 and 3 integrons among MDR *Salmonella* isolates from a selection of chicken broilers in the north of Iran.

## MATERIALS AND METHODS

### Bacterial strains.

All isolates used in this study were selected from a collection of *S.* Infantis strains obtained in our previous studies (14). *Salmonella* isolates were cultured on McConkey and *Salmonella* Shigella agar (MerckGermany) overnight at 37°C. To serotype, the isolates were tested using antisera O (B, D, C1 to C4) and H (Difco, USA) based on slide and tube agglutination tests.

Antibiotic susceptibility testing was performed using disc diffusion method (Kirby-Bauer) on Mueller-Hinton agar containing 14 antimicrobial disks [gentamicin ((GM: 10 μg), trimethoprim-sulfametoxasol (SXT: 5μg), nalidixic acid (NA: 30 μg), ciprofloxacin (CRO: 30μg), cefotaxime (CTX: 30 μg), imipenem (IPM: 10 μg), colistin (CL: 10 μg), ceftazidime (CAZ: 30 μg), amoxicillin (AMX: 30 μg), ampicillin (Amp: 10 μg), chloramphenicol(C: 30 μg), streptomycin (S: 10 μg), and tetracycline (TE: 30μg)) (MAST, UK). Results were interpreted according to the Clinical and Laboratory Standards Institute (CLSI) performance standards (CLSI, 2016). Isolates displaying resistance to 3 or more classes of antimicrobial disks were defined as multidrug-resistant (MDR). *Escherichia coli* ATCC 25922 was used as a reference strain for antibiotic disc control. Pure colonies of each isolate were collected in 2 sterile 1.5 microtubes, one tube containing 1 mL distilled water and the other containing physiological serum. Samples were kept at −20°C until DNA extraction.

### DNA extraction for PCR assay.

DNA was extracted from the isolates using purification Kit (Roche Applied Science, Mannheim, Germany) according to the manufacturer’s instructions. The DNA pellet was suspended in 100 μL of 10 mM TE buffer and stored at −20°C until use. DNA concentration and purity were assessed by Nano Drop 2000c spectrophotometer. A260/A280 values and concentration were confirmed by visualization on 1% agarose gel.

### Detection of antimicrobial resistance genes and class 1, 2 and 3 integrons.

*Salmonella* isolates, which showed resistance to each category of antimicrobial agents, were examined for the presence of resistance genes. The presence of genes associated with tetracyclines (*tetA, tetB* and *tetG*), chloramphenicol (*cat1* and *floR*), and sterptomycin (*strA*) were assessed by PCR.

PCR was performed using genus-specific primers *fljB* (Flagellar gene for *S*. Infantis) according to Kardos et al. (15). Primer sequences are presented in Table 1. To evaluate the specificity of the primers, we used *S.* Infantis (ATCC 51741) as positive control (provided from Persian Type Culture Collection of Iranian Research and scientific organisation), while sterile distilled water was used as negative control.

**Table 1:** Primers and their annealing temperatures used in this study

**Gene**	**Primer Sequence (5′→3′)**	**Size of product (bp)**	**Annealing temp.**	**Reference**
*fljB*	F: TTGCTTCAGCAGATGCTAAG	413	50°C	15
	R:TTGCTTCAGCAGATGCTAAG			
*Int1*	F:CAG TGG ACA TAA GCC TGT TC	164	55°C	16
	R: CCC GAG GCA TAG ACT GTA			
*Int2*	F: TTATTGCTGGGATTAGGC	233	58°C	17
	R: ACGGCTACCCTCTGTTATC			
*Int3*	F: AGTGGGTGGCGAATGAGTG	600	50°C	17
	R: TGTTCTTGTATCGGCAGGTG			
*strA*	F: CCAATCGCAGATAGAAGGC	548	50°C	18
	R :CTTGGTGATAACGGCAATTC			
*tetA*	F :GTAATTCTGAGCACTGTCGC	950	57°C	19
	R: CTGCCTGGACAACATTGCTT			
*tetB*	F :CTCAGTATTCCAAGCCTTTG	414	62°C	18
	R :ACTCCCCTGAGCTTGAGGGG			
*tetG*	F:GCAGCGAAAGCGTATTTGCG	680	62°C	20
	R :CCGAAAGCTGTCCAAGCAT			
*floR*	F: ATGGCAGGCGATATTCATTA	548	55°C	6
	R: AAACGGGTTGTCACGATCAT			
*cat1*	F: AACCAGACCGTTCAGCTGGAT	549	55°C	21
	R: CCTGCCACTCATCGCAGTAC			

PCR reaction mixture consisted of 2.5 μL 10× amplification buffer [500 mM KCl, 100 mM Tris/HCl (pH 8.5), 1.0% Triton X-100], 0.5 μL 25 mM MgCl_2_, 0.3 μL each 2.5 mM dNTPs (Fermentas, GmbH, Germany), 0.5 μL forward and reverse primers (20 ng/μl), 0.2 μL Taq DNA polymerase (5 U/μL), and 5 μL extracted DNA.

The cycling conditions used in PCR consisted of an initial denaturation step (95°C, 5 min), followed by 35 cycles of denaturation (95°C for 1 min), annealing of primers (58°C for 1 min), extension step (72°C, 2 min), and a final extension step at 72°C for 10 minutes. The amplified PCR products were analyzed by electrophoresis on 1.5% agarose gel stained with 0.5 μg/mL of ethidium bromide and visualized and confirmed under UV transilluminator.

Also, the presence of class 1, 2 and 3 integrons was tested in all MDR isolates using specific primers for integrase genes of integrons *intI1, intI2* and *intI3*. Primer sequences (6, 16–21) and the size of PCR products are shown in Table 1. To ensure the reliability of the results, all the samples were processed in duplicate.

Selected PCR products representing different amplicon sizes were extracted from the bands of the gel using a gel extraction kit (Qiagen, GmbH, Germany) and were evaluated by sequencing in both directions with the same PCR primers in 10 μL reactions. Sequencing results were analyzed using the GenBank database of the National Center for Biotechnology Information via the Basic Local Alignment Search Tool (BLAST) network service to understand the nature of resistance gene cassette. GenBank accession number KM659391 was used to determine the sequence identity class 2 integrons genes.

### Statistical analysis.

The collected data were statistically analyzed using SPSS program (software Version 17.0). Generated data were subjected to descriptive statistics and expressed in percentages.

## RESULTS

All isolates used in the study were confirmed as *Salmonella* by PCR amplification of the *fljB* gene, which produced 413bp amplicons.

**Fig. 1:**
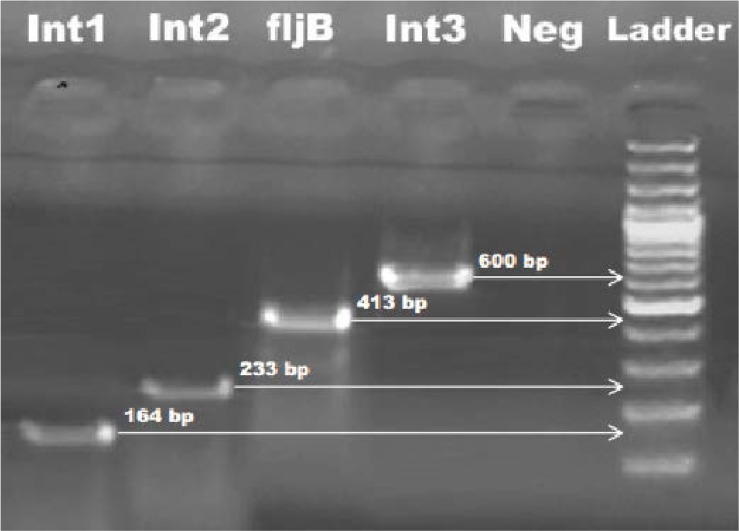
PCR assays for identification of Integrone and *floR* gene. DNA size marker (100 bp DNA ladder)

Class 1 integrons were found among 18 resistant isolates (36%) in our samples (16), class 2 integrons were observed in 21 (42%) isolates, 11 (22%) of the isolates carried both classes, and 2 (4%) isolates harbored class 3 integrons (Fig. 1).

Nalidixic acid and trimethoprim resistant isolates were the most common in harboring class 1 and 2 integrons. The relationship between antibiotic resistance and existence of different integrons is demonstrated in Table 2.

**Table 2:** Distribution of *intI1* and *intI2* among *S*. Infantis isolates resistant to different antibiotic agents

**Antibiotic agent**	**Number of resistant isolate**	**Class 1 integron n (%)**	**Class 2 integron n (%)**	**Class 1 & 2 integron n (%)**
Ceftazidim	10	7 (70)	7 (70)	4(40)
Nalidixic acid	50	18 (36)	21 (42)	11(22)
Tetracyclin	47	16 (34)	19 (40)	10(21)
Sterptomycin	43	16 (37)	20 (46)	10(23)
Chloramphenicol	36	12 (33)	15 (41)	5(13.8)
Trimethoprim	50	18 (36)	21 (22)	11(36)

**Fig. 2:**
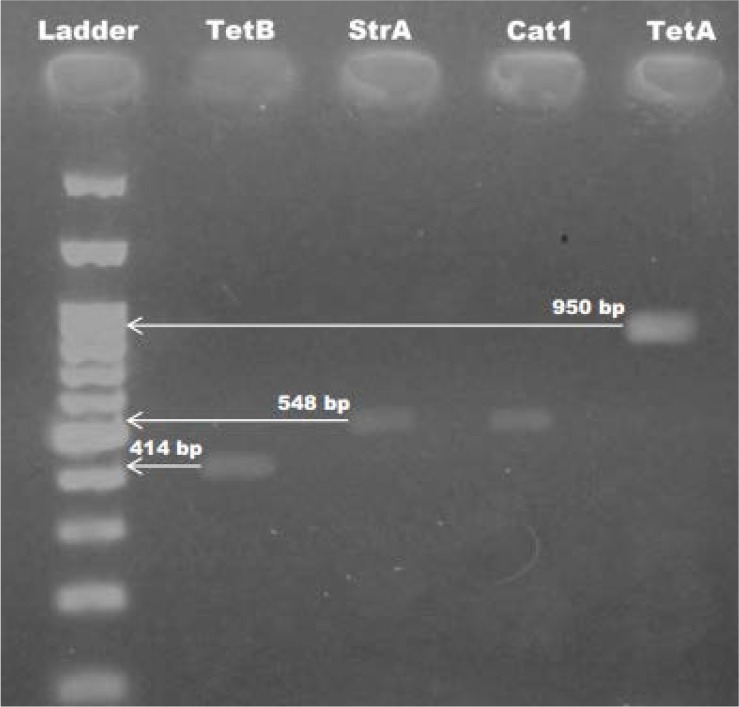
Detecton of **tet A, tetB, cat1** and *strA* gene amplicons by agarose gel electrophoresis

Antimicrobial resistance genes were detected in all *S.* Infantis isolates. PCR results indicated that all 47 tetracycline-resistant strains carried the *tetA* gene and 5 (10.6%) of the isolates carried the *tetB* gene (Fig. 2). However, none of *S.* Infantis isolates showed the *tetG* gene.

Nine (18%) of the *S.* Infantis isolates harbored the *strA* gene, conferring resistance to sterptomycin. All 36 chloramphenicol-resistant isolates carried the *floR* and *cat* genes with the expected bands (Fig. 2). Detailed data of antimicrobial resistance and the resistance gene profiles of *S.* Infantis are demonstrated in Table 3.

**Table 3:** Antimicrobial resistance and resistant gene profiles of *S.* Infantis isolates

**Isolates**	**Antimicrobial resistance pattern**	**Antimicrobial-resistant genes**
17, 23, 55	CAZ, NA, C, SXT, St, TE	*Int1, tetA, cat1, flor*
18, 100	CAZ, NA, C, SXT, St, TE	*tetA, cat1, flor*
89, 96, 97	CAZ, NA, C, SXT, St, TE	*Int1, Int2, strA, tet A, cat1, flor*
93	CAZ, NA, C, SXT, St, TE	*Int2, tetA, cat1, flor*
95	CAZ, NA, C, SXT, St, TE	*Int1, Int2, tetA, cat1, flor*
1,65	CAZ, NA, C, SXT, St, TE	*Int2, tetA, cat1, flor*
24, 45, 82	NA, C, SXT, St, TE	*Int2, tetA, cat1, flor*
34	NA, C, SXT, St, TE	*Int1, Int3, tetA, cat1, flor*
36	NA, C, SXT, St, TE	*Int3, tetA, cat1, flor*
42	NA, C, SXT, St, TE	*tetA, tetB, cat1, flor*
10, 33, 51, 67, 92, 11	NA, C, SXT, St, TE	*tetA, cat1, flor*
59	NA, C, SXT, St, TE	*Int1, tetA, tetB, strA, cat1, flor*
60	NA, C, SXT, St, TE	*Int2, tetA, strA, cat1, flor*
63	NA, C, SXT, St, TE	*tetA, strA, cat1, flor*
69, 99	NA, C, SXT, St, TE	*Int1, Int2, tetA, cat1, flor*
85	NA, C, SXT, St, TE	*tetA, cat1, flor*
87	NA, C, SXT, St, TE	*tetA, tetB, cat1, flor*
6	NA, C, SXT, St, TE	*Int1, tetA*
8, 22	NA, C, SXT, TE	*tetA, cat1, flor*
3, 77, 79	NA, SXT, St, TE	*tetA*
39	NA, C, SXT, St	*Int1, Int2, tetA, cat1, flor*
21, 13	NA, SXT, St, TE	*Int1, Int2, tetA*
2, 5	NA, SXT, St, TE	*Int1, Int2, strA, tetA*
4	NA, SXT, St, TE	*Int2, tetA*
9	NA, C, SXT, St	*Int2, tetA, tetB, cat1, flor*
12, 7	NA, SXT, TE	*tetA*
15	NA, SXT, TE	*tetA, tetB*
16	NA, SXT, TE	*tetA, strA*
32	NA, SXT, TE	*Int1, Int2, tetA*

## DISCUSSION

Antimicrobial-resistant *Salmonella* is an accepted global health problem. The increase in antimicrobial resistance is due to genetic mutations or horizontal and vertical transfer of genetic elements (22). *Salmonella* isolates used in the current study illustrated a high rate of multidrug resistance to more than 3 antibiotics. Our findings show that most of the tested antimicrobial resistance genes showed high rates of resistance, indicating that these genes play an important role in drug resistance among *Salmonella* isolates. Several studies have focused on investigating the connection between the presence of integrons and resistance genes in multidrug resistance *Salmonella* strains in different countries (23, 24). Class 1 and 2 integrons are commonly observed among MDR isolates, so they are usually referred to as MDR integrons (25). While the frequency of class 1 integrons remained stable over time (16), our study confirmed a slight increase in the presence of class 2 integrons (42%) in *S*. Infantis isolates. The results of this study revealed that class 1 and class 2 integrons differ in their behavior as MDR markers, which is similar to the reports of other studies (6, 26–27).

Tetracycline is commonly used as an antimicrobial agent in human and veterinary medicine. Incidences of tetracycline resistance have been described recently in Iran and other countries (28, 29). However, in *Salmonella* spp. isolates, tetracycline resistance is usually mediated by the following determinants: *tetA, tetB, tetC, tetD* and *tetG* (30, 31). The tet-resistant genes occurred most frequently in our study. The prevalence of *tetA* was higher than *tet B* in the screened isolates. No isolates carried *tetG.* Several studies reported the range of *Salmonella* carrying the tetracycline resistance gene *tetA* to be 60% to 100% (32–34). *tetA* and *tetB* are located inside non-conjugative transposons; this is an important method for the horizontal transfer of antibiotic resistance (35). The present study showed the detection of *cat1* and *floR* gene in all chloramphenicol resistant isolates. Chloramphenicol is used for the treatment of salmonellosis in animals, but it seems that resistance to this antibiotic is increasing. The *cat1* gene, which encodes chloramphenicol acetyl transferase, was detected in all the chloramphenicol resistant isolates harboring the *floR* gene. In other studies conducted on *S*. *infantis*, the *cat1* gene was predominately observed in chloramphenicol-resistant isolates (15, 36–37). This study revealed a high frequency of antimicrobial resistance genes among *Salmonella* isolates from chicken broilers, which is extensively spread in the north of Iran. Our results suggest that integrons are common among MDR isolates and they can be used as a marker for the identification of MDR isolates. Therefore, public health professionals should use some management strategies to prevent food-borne diseases caused by MDR *Salmonella* in the food supply.
